# Chemical composition and the insecticidal activity of *Aeollanthus pubescens* leaf essential oil against *Anopheles gambiae* sensu stricto

**DOI:** 10.1186/s13071-021-05012-w

**Published:** 2021-10-07

**Authors:** Roméo Barnabé Bohounton, Luc Salako Djogbénou, Oswald Yédjinnavênan Djihinto, Oronce Sedjro-Ludolphe Dedome, Pierre Marie Sovegnon, Bruno Barea, Aristide Adomou, Pierre Villeneuve, Fidèle Paul Tchobo

**Affiliations:** 1 Laboratory of Study and Research of Applied Chemistry, Polytechnic School of Abomey-Calavi, Cotonou, Benin; 2 Tropical Infectious Diseases Research Centre (TIDRC), University of Abomey Calavi, Abomey-Calavi, Benin; 3 Department of Vector Biology, Liverpool School of Tropical Medicine, Pembroke Place, Liverpool, UK; 4 Laboratoire de Botanique Et Écologie Végétale (LaBEV), Faculté Des Sciences Et Techniques (FAST), University of Abomey-Calavi, Cotonou, Benin; 5 CIRAD-PERSYST-UMR IATE, Montpellier, France

**Keywords:** *Aeollanthus pubescens*, *Anopheles gambiae*, Essential oil, Bioinsecticidal activity

## Abstract

**Background:**

The excessive use of synthetic insecticides is responsible for many cases of resistance in insects. Therefore, the use of natural molecules of ecological interest with insecticidal properties is an alternative approach to the use of synthetic insecticides. The aim of this study is to investigating the larvicidal and adulticidal activity and the chemical composition of the essential oil of *Aeollanthus pubescens* on the major malaria vector, *Anopheles gambiae*.

**Methods:**

Three reference strains of *Anopheles gambiae* sensu stricto (Kisumu, Kiskdr and Acerkis) were used in this study. The leaves of *A. pubescens* were collected in southern Benin. The standard World Health Organisation (WHO) guidelines for larvicide evaluation were used, and the chemical composition of the essential oil was analysed by gas chromatography coupled to mass spectrometry. Adult mosquitoes of each strain were exposed to pieces of net coated with the essential oil for 3 min using the WHO cone bioassay method. Probit regression analysis was used to determine the concentrations that would kill 50 and 95% of each test population (LC_50_, LC_95_) and the knockdown time for 50 and 95% of each test population (KDT_50_, and KDT_95_). The difference between the mortality–dose regressions for the different strains was analysed using the likelihood ratio test (LRT). The log-rank test was performed to evaluate the difference in survival between the strains.

**Results:**

A total of 14 components were identified, accounting for 98.3% of total oil content. The major components were carvacrol (51.1%), thymyle acetate (14.0%) and ɣ-terpinene (10.6%). The essential oil showed larvicidal properties on the Kisumu, Acerkis and Kiskdr strains, with LC_50_ of 29.6, 22.9 and 28.4 ppm, respectively. With pieces of netting treated at 165 µg/cm^2^, the KDT_50_ of both Acerkis (1.71 s; *Z* = 3.34, *P* < 0.001) and Kiskdr (2.67 s; *Z* = 3.49, *P* < 0.001) individuals were significantly lower than that of Kisumu (3.8 s). The lifespan of the three mosquito strains decreased to 1 day for Kisumu (*χ*^2^ = 99, *df* = 1, *P* < 0.001), 2 days for Acerkis (*χ*^2^ = 117, *df* = 1, *P* < 0.001) and 3 days for Kiskdr (*χ*^2^ = 96.9, *df* = 1, *P* < 0.001).

**Conclusion:**

Our findings show that *A. pubescens* essential oil has larvicide and adulticide properties against the malaria vector *An. gambiae* sensu stricto, suggesting that this essential oil may be a potential candidate for the control of the resistant malaria-transmitting vectors.

**Graphical Abstract:**

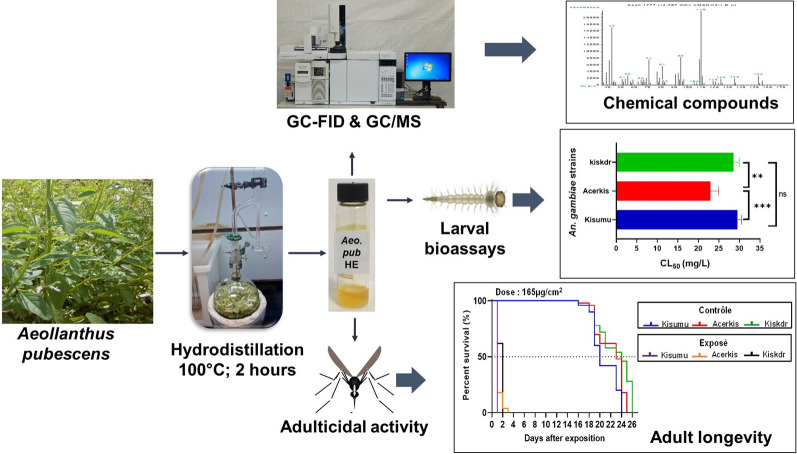

**Supplementary Information:**

The online version contains supplementary material available at 10.1186/s13071-021-05012-w.

## Background

Vector-borne diseases remain the major causes of death in many tropical countries. The most important vector-borne diseases are malaria, lymphatic filariasis, dengue fever and yellow fever, and all of the pathogens responsible for these diseases are transmitted by mosquitoes [1, 2]. Among mosquito-borne infectious diseases, malaria is one of the most deadly, and it is a major focus of public health concern in terms of incidence, prevalence, morbidity and mortality in low-income countries of Africa, Asia and Latin America [3, 4]. Despite efforts of the respective national malaria control programmes, nearly 85% of malaria deaths occurred in 21 sub-Saharan African countries, including the Republic of Benin [4]. Malaria is transmitted through the bites of parasite-infected *Anopheles* female mosquitoes [5]. In the Republic of Benin (West Africa), *Anopheles arabiensis*, *Anopheles coluzzii* and *Anopheles gambiae* are the major malaria vectors [6, 7], but *An. gambiae* is the most prevalent vector across the country regardless of the three differentiated bioclimates present (Guinean-bioclimatic zone in the south; tropical Sudano-Guinean climate in the center; a Sudanian semi-dry bioclimate in the north) [6].

So far, most malaria control programmes have mainly relied on artemisinin-based combination therapies (ACTs) for the treatment of diagnosed patients and the use of chemical compounds, through insecticide-treated nets (ITNs) and indoor residual spraying (IRS), for the prevention of human–vector contact [8]. The World Health Association (WHO) Pesticide Evaluation Scheme (WHOPES) currently recommends 14 insecticides belonging to five major classes of synthetic chemical for IRS [9] and insecticides from the pyrethroid group for ITNs [10]. However, the overuse of these synthetic chemical insecticides has led to the emergence of resistant malaria vectors, and the frequency of insecticide resistance is widespread, especially in African regions [11, 12]. Also, these synthetic insecticides have been recognised to have adverse effects on non-targeted species and affect animal and plant biodiversity [13–15]. Their indiscriminate use has also been shown to have severe effects on the environment and impacts on human health [16].

These findings combined with clear indications of multidrug resistance in the malaria parasite [17] and the absence of an effective vaccine have led scientists to focus on searching for environmentally-friendly vector control alternatives with the aim of decreasing the selection pressure for insecticide resistance [18]. These kinds of eco-friendly vector control alternatives could be achieved especially with insecticides from botanical sources, which have the potential to be safer for humans and the environment and have a minimal residual effect [19]. Compared to their synthetic counterparts, natural insecticides are more target specific, less toxic to vertebrates and more sustainable than their synthetic counterparts [19, 20].

Beninese traditional medicine and pharmacopoeia medications are richly bio-diversified, which could be a major source of natural insecticides for malaria control [21]. Therefore, it appeared of interest to learn more about Beninese flora with regards to their insecticidal activities. However, to our knowledge, few studies have been conducted on the mosquitocidal activity of extracts from Beninese plant species [22–24].

*Aeollanthus pubescens* (common name in Benin: Iko) is an annual herbaceous plant belonging to family Lamiaceae family that is found across Benin and distributed in many West African countries [25]. It is commonly used by local populations as food (spice or green vegetables) and medicine (treatment of diarrhea, fever, haemorrhage, upper respiratory tract contaminations) [26, 27]. In Benin, this plant develops for the most part in the rocky regions found in the center of the country. Previous work has already demonstrated the insecticidal activity of *A. pubescens* essential oil against the coffee berry borer, *Hypothenemus hampei*, a significant coffee pest with a global distribution [28]. However, there is no report so far in the literature on the insecticidal properties of *A. pubescens* essential oils against malaria vectors. The aim of this study was to investigate the larvicidal and adulticidal activity and the chemical composition of the essential oil of *A. pubescens* under laboratory conditions, with the ultimate aim of identifying safer alternatives to the existing synthetic insecticides for combating malaria vector *Anopheles gambiae* sensu stricto (s.s.).

## Methods

### Plant material and extraction

The leaves of *A. pubescens* Benth were collected in July 2014 in Covè, Benin (7°28′25.2″N, 2°19′13.0″E) and authenticated at the National Herbarium of University of Abomey-Calavi (UAC) where they were kept under voucher code AAC 188/HNB.

The leaves were shade dried at 25 °C ± 2 °C for 72 h. Three batches of 200 g of dried leaves were submitted to hydro-distillation in a Clevenger apparatus at 100 °C for 2 h. The distilled oil was dried using anhydrous sodium sulphate and transferred into an airtight amber-coloured vial and stored at 4 °C until further use. The yields were averaged over the three experiments of the plant materials.

### Chemical analysis of the essential oil of *A. pubescens* leaves

#### Analysis by gas chromatography coupled with flame ionisation detection

Both The gas chromatography coupled with flame ionisation detection (GC–FID) and GC coupled with mass spectrometry (GS–MS) methods described by Tchobo et al. [29] were used with slight modifications. The essential oil constituents were analysed by a capillary GC-FID equipped with a Supelco SPB-1 column (internal diameter [i.d.]: 30 m × 0.32 mm ; film thickness: 0.25 µm) (Supelco Inc., Sigma-Aldrich, Bellefonte, PA, USA). A 1-µl sample of the essential oil diluted in chloroform was directly injected into the GC system. Helium was used as carrier gas at a flow rate was 6 ml/min, and the splitting ratio was 1/17. The inlet temperature profile was 250 °C/min, 200–310 °C at 20 °C/min and then maintained at 310 °C for 2 min.

A capillary GC–MS was used on a TR-1MS column (i.d.: 30 m × 0.25 mm ; film thickness: 0.25 µm) (Thermo Fisher Scientific, Waltham, MA, USA). An electron impact system was used with ionisation energy of 70 eV. Helium was used as the carrier gas at a flow rate of 0.6 ml/min, and the splitting ratio was 1/17. The temperature settings were: 70–200 °C at 10 °C/min, 200–300 °C at 20 °C/min and then maintained at 300 °C for 1 min. Inlet and MS transfer line temperatures were set at 250 °C and 320 °C, respectively. All apparatus and accessories were from Thermo Fisher Scientific, as were all chromatography data system software (Chromocard and XCalibur). Identification of the essential oil constituents was based on the comparison of their retention times and their Kovats retention indexes relative to (C_8_–C_20_) n-alkanes. Whenever possible, identifications were based on mass spectra of the authentic standard compounds; otherwise, identifications were performed using published data [30] and comparison with the National Institute of Standards and Technology (NIST) mass spectral library.

### Mosquito strains

Three *An. gambiae* s.s. laboratory strains (Kisumu, Acerkis, Kiskdr) that were regularly maintained at the insectary of the laboratory of Vector-Borne Infectious Diseases at the Institut Régional de Santé Publique Alfred Quenum (IRSP-AQ) of the University of Abomey-Calavi in Ouidah (Benin) were used in this study. The Kisumu strain originates from Kenya and is a reference strain susceptible to all insecticides [31]. The Acerkis strain is resistant to both organophosphate and carbamate insecticides and is homozygous for the G119S mutation [32]. The Kiskdr strain is homozygous for the knockdown resistance (*kdr*^*R*^) allele (L1014F) that confers resistance to pyrethroids and dichlorodiphenyltrichloroethane (DDT) [33]. Both the Acerkis and Kiskdr strains are assumed to share the same genetic background as the Kisumu strain but differ by the presence of resistance alleles.

The colonies of the three strains were maintained at the insectary under optimum conditions (25–27 °C and 70–80% relative humidity). The third instar larvae and 3- to 5-day-old adult females from generation 42 (G42) of each mosquito strain were used for the bioassays.

### Bioassays

#### Larval bioassay

The larvicidal properties of the essential oil were assayed according to the standard method recommended by the WHO [34] with slight modifications. Since the essential oil does not dissolve in water, six different concentrations (1000, 2000, 3000, 4000 and 5000 ppm) of the essential oil were prepared in 96% ethanol. For this bioassay, 25 third-instar larvae of each strain were gently transferred into a plastic beaker containing 99 ml of water, following which 1 ml of each prepared concentration was added to obtain test solutions of 10, 20, 30, 40 and 50 ppm. During the bioassays, larvae were exposed for 24 h at 26 ± 2 °C (temperature measured using Waranet kit; Waranet Solutions SAS, Auch, France) without any food. After exposure, larval mortality was recorded. Larvae were considered dead when they were not able to move or swim actively when touched. For each strain, four replicates were performed for a total of 100 larvae per concentration. The control group consisted of batches of larvae exposed to water and the ethanol solvent alone. In total, three different experiments were conducted on three different days.

#### Adult Bioassay

Fragments of insecticide-free netting (13 × 13 cm; 169 cm^2^) were coated with the essential oil using a method described by Aurelie et al. [35]. The mass of essential oil proportional to the net area (169 cm^2^) per concentration was determined: 9.3, 18.6 and 27.9 mg to achieve impregnation at 55, 110 and 165 µg/cm^2^, respectively, after preliminary doses screening. A 1.5-ml aliquot of ethanol (high-perfomance liquid chromatography grade) was poured into a Petri dish containing the mass of essential oil corresponding to a given concentration. After complete dissolution, the fragment of the mosquito net was coated with the mixture. The impregnated net pieces were left to dry at room temperature for 5 min to allow the essential oil to adhere to the mosquito net and to completely evaporate the ethanol. After drying, treated net pieces were maintained in the dark to prevent likely reactions of the essential oil constituents with the light and stored at 4 °C for 2–4 h until the time to perform the cone tests. All coated net pieces used during the day were treated in the morning at the same time. Different coated net pieces were used in each replicate to avoid any loss of concentration of the essential oil. Pieces of nets of the same size were also treated with 1.5 ml of ethanol and used as controls.

**Fig. 1:**
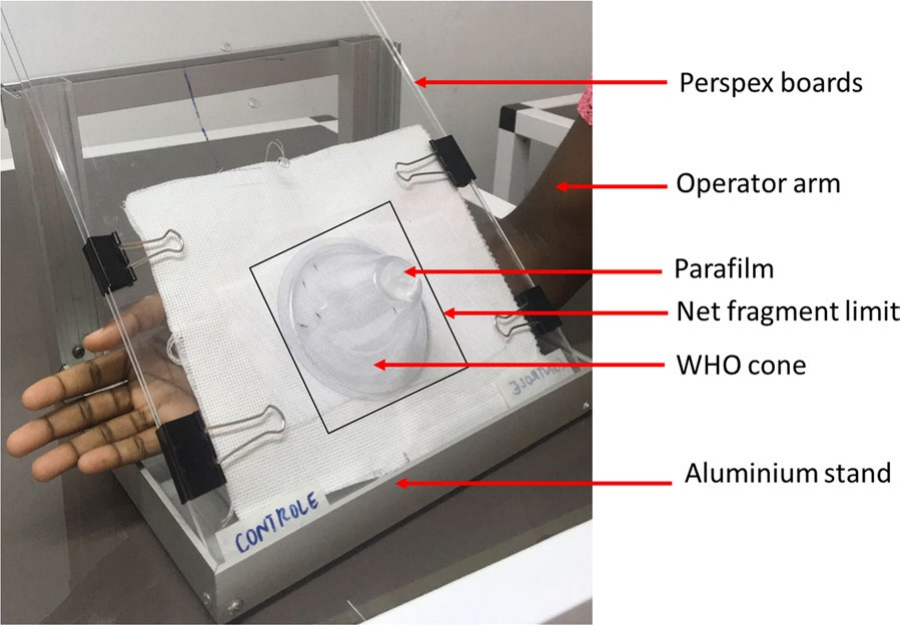
Cone test experimental set-up

The cone test was used to assess the adulticidal activity of the essential oil on the adult mosquitoes. The cone test is an adaptation of the WHO cone bioassay [36], with the following modification: during the assay, the test operator held a forearm behind the cone to provide a host for attraction (Fig. 1).

Unfed 3- to 5-day-old female mosquitoes of the Kisumu, Acerkis and Kiskdr strains were used in the test. On the day of testing, females were starved for 4 h before testing. Groups of five female mosquitoes were placed into plastic cups and moved into the testing room 1 h before testing began to allow the mosquitoes to acclimatise to room conditions. The pieces of net, test or control pieces, were placed over a hole on the Perspex boards and secured using a clear tape. A second Perspex board was laid on the first board creating a test/control net “sandwich” between the two boards. The cone was placed over the net and sealed at the top with a piece of parafilm. A batch of five mosquitoes was transferred into the cone with the operator’s forearm in position. Mosquitoes were then kept in the cones for 3 min. Ten replicates of batches of five mosquitoes of each strain were tested per concentration of impregnated nets.

### Monitoring of the lethal effect of mosquito exposure to the essential oil

After exposure, mosquitoes were removed from the cone, transferred into a recovery cups and provided with 10% honey solution soaked on a cotton pad. Mosquito knockdown was recorded at 60 min post-test. Mosquito mortality was then recorded every day until the death of the last female of each mosquito strain.

### Data analysis

The analysis of dose–mortality responses in the larval bioassays was performed using the BioRssay script version 6.2 [37] in R software version 3.0 [38]. This script calculates the mortality–dose regression using a generalised linear model (GLM). To assess the adequacy of the model, a chi-square (*χ*^2^) test between the observed dead numbers (data) and the dead numbers predicted by the regression was used; this also tests whether the mortality–dose regressions were similar for the different strains, using a likelihood ratio test (LRT). If more than two strains are tested, it also computes the pairwise test and corrects it using sequential Bonferroni correction [37]. Finally, it computes the lethal concentrations inducing 50 and 95% mortality (LC_50_ and LC_95_, respectively) recorded in each strain and the associated confidence intervals (CI), and the 50 and 95% resistance ratios (RR_50_ and RR_95_, respectively; i.e. the LC_50_ or LC_95_ in each strain divided by the LC_50_ or LC_95_, respectively, of the reference strain) and their 95% CI. Susceptible or resistant status was defined according to Mazzarri and Georghiou [39] and Bisset et al. [40] criteria: RR_50_ ≤ 1 indicates susceptibility to the tested insecticide, while RR_50_ > 1 indicates insecticide resistance. Three categories of resistance levels were assigned: low resistance (RR_50_ < 5), moderate resistance (5 ≤ RR_50_ ≤ 10) and high resistance (RR_50_ > 10) [39, 40]. The times at which 50 or 95% of mosquitoes fell on their back or their side, taken to be the knockdown time (KDT_50_ or KDT_95_, respectively), and their 95% CI were estimated after probit regression in R software using the package ‘ecotox’ [41] based on the method described by Finney [42]; the difference between two KDT_50_ values was tested using the ratio test developed by Wheeler et al. [43]. Mosquito survival after exposure to the essential oil-impregnated net was analysed by Kaplan–Meier survival curves using GraphPad Prism 8.0.2 software (GraphPad Software, San Diego, CA, USA). The log-rank test was performed to evaluate the difference in survival between the strains. All statistical analyses were set at a significance threshold of *P* < 0.001.

## Results

### Chemical composition of *A. pubescens* leaf essential oil

The percentage yields of essential oil obtained from the hydro-distillation of the leaves of *A. pubescens* was 0.3 ± 0.02% [w/w based on fresh leaves; mean ±standard error (SE)]. Analysis of the chemical composition of the essential oil of *A. pubescens* (Table 1) identified 14 compounds, accounting for 98.3% of the crude essential oil’s mass. The essential oil of *A. pubescens* aerial part had a higher content of (60.4%) than monoterpene hydrocarbons (22.4%) and sesquiterpene hydrocarbons (15.5%) (Table 1). The major component of the essential oil was carvacrol (51.1%), followed by other components such as thymol acetate (14.0%), ɣ-terpinene (10.6%),* O*-cymene (8.4%) and thymol (5.5%). The percentage of composition of the remaining nine compounds ranged from 0.2 to 2.0% (Table 1).

**Table 1:** Chemical composition of the *Aeollanthus pubescens* essential oil

Peak no	RI^a^	Components	Peak area (%)
1	940	α-Pinene	0.6
2	986	Myrcene	2.0
3	1005	Lumicolchicine	0.2
4	1020	*O*-Cymene	8.4
5	1031	1,8-cineole	0.6
6	1057	γ-Terpinene	10.6
7	1088	Linalool	0.9
8	1162	Borneol	1.4
9	1173	Terpin-4-ol	1.6
10	1273	Thymol	5.5
11	1284	Carvacrol	51.1
12	1359	Thymol acetate	14.0
13	1488	β-Cubebene	0.2
14	1503	Acid [(2,4,6-triethylbenzoyl) thio] acetic	1.3
		Total identified (%)	98.3
		Sesquiterpenes hydrocarbons	15.5
		Monoterpenes hydrocarbons	22.4
		Oxygenated monoterpenes	60.4

^a^RI refers to the relative retention index, as determined on an HP-1 column using the homologous series of n-alkanes

### Toxicity of *A. pubescens* essential oil on *An. gambiae* s.s. larvae

Larval bioassays conducted on *An. gambiae* larvae showed that *A. pubescens* essential oil had considerable larvicidal activity, with LC_50_ values of 22.9, 28.5 and 29.6 ppm for essential oil from the Acerkis, Kiskdr and Kisumu strains, respectively (Table 2) (Additional file 1: Table S1). No mortality was recorded in the control batches of each strain treated with ethanol. The chi-square test between the observed dead numbers (data) and the dead numbers predicted by the log-dose probit-mortality regression indicated that the data were well fitted by a straight line (Table 2). Acerkis and Kiskdr strains were both susceptible to the essential oil, with lower RR_50_ values (0.8 and 0.9 for the Acerkis and Kiskdr strains, respectively; Table 2). The LRT showed that Acerkis strain larvae (LC_50_ = 22.9 ppm) were significantly more susceptible to the essential oil than Kiskdr (LC_50_ = 28.5 ppm; *P* < 0.001) and Kisumu larvae (LC_50_ = 29.6 ppm; *P* < 0.001) (Table 2). However, the susceptibility of Kisumu and Kiskdr larvae did not significantly differ (*P* = 0.41).

**Table 2:** Toxicity of *A. pubescens* against *Anopheles gambiae* sensu stricto larvae after 24 h of exposure

Mosquito strains	LC_50_ (ppm)	95% CI	RR_50_	95% CI ( LCL-UCL)	LC_95_ (ppm)	95% CI (LCL–UCL)	Slope (± SE)	Intercept (± SE)	Chi(*P*) value^a^
Kisumu	29.6	28.6–30.6	–	–	49.9	47.4–53.2	7.2 ± 0.4	− 10.6 ± 0.5	0.9
Acerkis	22.9	20.9–24.9	0.8	0.6–0.9	52.3	46.1–61.8	4.6 ± 0.4	− 6.3 ± 0.5	0.3
Kiskdr	28.5	27.1–29.9	0.9	0.8–1.2	49.7	46.1–54.9	6.8 ± 0.5	− 9.9 ± 0.7	0.1

No mortality was observed in the control group of each mosquito strain

*LC*_*50*,_,* LC*_*95*_ Lethal concentrations (50 and 95% mortality, respectively), *C.I* confidence interval, *RR*_*50*_ resistance ratio at LC_50_: LC_50_ [resistant strain)/ LC_50_ (Kisumu)], *LCL* lower confidence limit, *UCL* upper confidence limit, *SE* standard error

^a^Chi(*P*) is indicated to judge whether the data are well fitted to the regression or not. The fits are acceptable when the *P*-value is > 0.05

### Adulticidal activity of *A. pubescens* essential oil against *An. gambiae* s.s. strains

#### Knockdown time

The average time estimated for KDT_50_ or KDT_95_ of adult *An. gambiae* females of each strain decreased with the increasing treatment concentration. The KDT_50_ was < 4 s for all mosquito strains in contact with the net pieces treated at 165 µg/cm^2^ (3.8 s for Kisumu; 1.7 s for Acerkis; 2.7 s for Kiskdr), which were significantly lower than that recorded at the lowest essential oil treatment (55 µg/cm^2^) (Kisumu: 22.1 s, *Z* = 30.09, *P* < 0.001; Acerkis: 291.7 s, *Z* = 22.07, *P* < 0.001; Kiskdr: 591.6 s, *Z* = 62.17, *P* = 0) (Table 3) (Additional file 1: Table S2). At the highest treatment concentration (165 µg/cm^2^), both Acerkis and Kiskdr mosquitoes were knocked down more quickly (Acerkis KDT_50_: 1.7 s, *Z* = 3.34, *P* < 0.001; Kiskdr KDT_50_: 2.7 s, *Z* = 3.49, *P* < 0.001) than Kisumu individuals (KDT_50_: 3.8 s). However, the highest knockdown times were observed for the Kiskdr (KDT_50_ > 597 s) and Acerkis (KDT_50_ > 291 s) females exposed to the essential oil at 55 µg/cm^2^.

**Table 3:** Times for 50 and 95% knockdown of *An. gambiae* sensu stricto strains per piece of treated net

Strain	Essential oil treatment (µg/cm^2^)	KDT_50_ (s)	95% CI ([LCL–UCL)	KDT_95_ (s)	95% CI ([LCL–UCL)	*χ*^2^ value of the Pearson goodness-of-fit test	Slope	Intercept
Kisumu	55	22.1	[20.0–23.8]	45.3	[40.5–53.3]	22.2	5.3	− 7.1
	110	4.7	[4.5–4.9]	6.3	[5.9–6.8]	2.2	13.5	− 9.1
	165	3.8	[3.6–3.9]	5.7	[5.2–6.4]	2.5	9.4	− 5.4
Acerkis	55	291.72	[280.6–302.4]	373.7	[355.5–400.7]	3.6	15.3	− 37.7
	110	4.6	[4.4–4.8]	6.2	[5.8–6.8]	2.4	23.7	− 15.8
	165	1.7	[1.5–1.8]	3.5	[3.1–4.3]	2.3	5.2	− 1.2
Kiskdr	55	591.6	[576.4–607.6]	813.0	[777.6–859.0]	10.6	11.9	0.8
	110	197.4	[185.0–209.3]	308.7	[285.8–341.9]	12.3	8.5	− 19.5
	165	2.7	[2.5–2.9]	4.4	[3.9–5.1]	1.7	7.6	− 3.2

The probit regressions parameters (*χ*^2^ value of the Pearson goodness-of-fit test, slope and intercept) are indicated. No knockdown time was recorded in the control group of each mosquito strain

*KDT*_*50*_,*KDT*_*95*_ Knockdown times for 50 and 95% of adult mosquitoes after 3 min of exposure to the net pieces impregnated with the essential oil in the cone test

#### Induced mortality

**Fig. 2:**
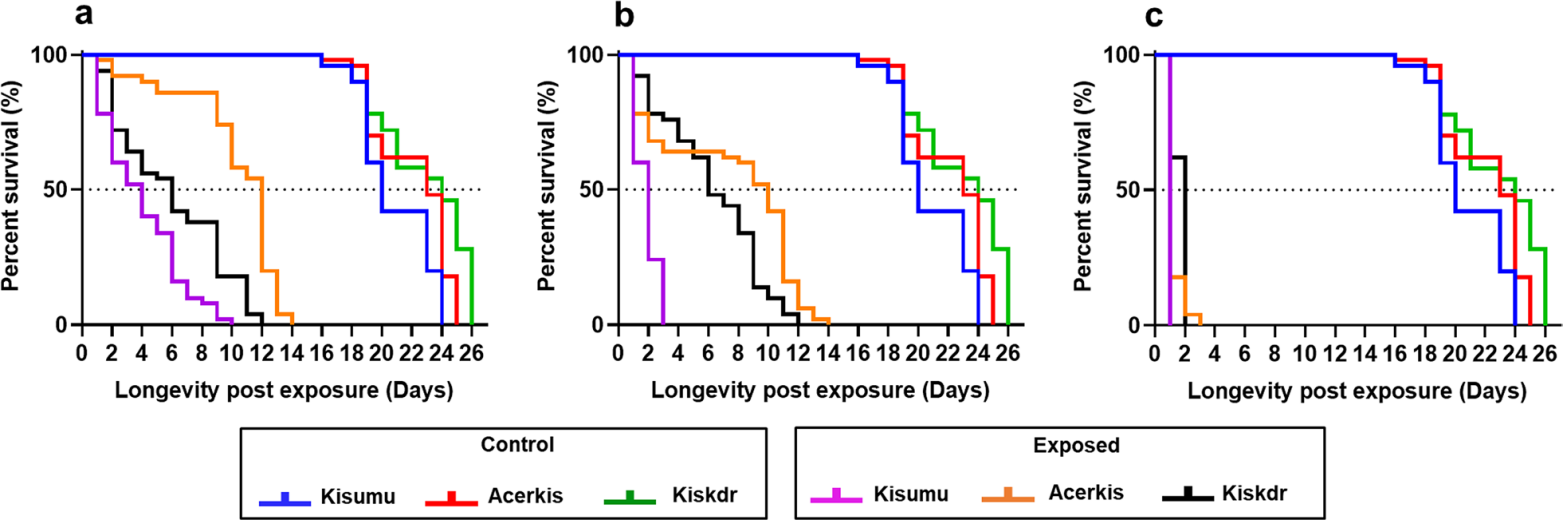
Survivorship of adult female mosquitoes post-exposure. Each mosquito strain was followed up after exposure to the net pieces impregnated with the *Aeollanthus pubescens* essential oil at 55 µg/cm^2^ (**a**), 110 µg/cm^2^ (**b**) and 165 µg/cm^2^ (**c**)

Overall, the three essential oil treatments (concentrations) significantly decreased the survival of all mosquito strains after exposure. For the essential oil coating at 165 µg/cm^2^, the longevity of the three mosquito strains decreased significantly from 24 days for Kisumu, 25 days for Acerkis and 26 days for Kiskdr in control groups to 1 day for Kisumu (*χ*^2^ = 99, *df* = 1, *P* < 0.001), 2 days for Acerkis (*χ*^*2*^ = 117, *df* = 1, *P* < 0.001) and 3 days for Kiskdr (*χ*^*2*^ = 96.9, *df* = 1, *P* < 0.001) in exposed groups (Fig. 2c) (Additional file 1: Table S3). With the net treated at 110 µg/cm^2^, the longevity of Kisumu females was significantly reduced by 21 days compared to that recorded with the 55 µg/cm^2^ treatment (by 14 days; *χ*^2^ = 28.6, *df* = 1, *P* < 0.001) (Fig. 2a, b). With each of these two treatments, no significant difference was observed on the longevity of Kiskdr (*χ*^2^ = 0, *df* = 1, *P* = 0.8).

## Discussion

The increasing number of reports of natural mosquito resistance to the existing synthetic insecticides has strengthened the focus on searching for environmentally-friendly insecticide compounds for vector control strategies. This is a beneficial alternative as essential oils represent a rich source of bioactive compounds that are biodegradable into non-toxic products and, due to their natural synergism, they reduce the risk of the development of resistance in the vectors [44]. In addition, essential oils are known to be nucleophilic in nature and efficiently hinder a range of biological processes (metabolic, physiological, biochemical and behavioural) in insects [45–47]. The present study is the first report of the larvicidal and adulticidal activity of *A. pubescens* leaves essential oil on the major African malaria vector *An. gambiae* s.s. The bioassays of the insecticidal properties of the essential oil of *A. pubescens* leaves were carried out in the laboratory using immature and adult stages of *An. gambiae* s.s.

The chemical analysis of the essential oil of *A. pubescens* revealed 14 compounds. Carvacrol was the major component, representing 51.1% of the total content, followed by the thymol acetate (14.0%) and γ-terpinene (10.6%). This oil composition is characteristic of the carvacrol chemotype. Overall, five different chemotypes were identified from the essential oil of *A. pubescens* aerial parts from Togo: (i) the thymol chemotype, with a thymol content of 46.3–58% of thymol; (ii) the carvacrol chemotype, with a carvacrol content of 58.2% carvacrol; (iii) the carvacrol and thymol chemotype, with a content of 41% carvacrol and 27% thymol; (iv) the carvacrol and thymol acetate chemotype, with a content of 55.4% carvacrol and 35.1% thymol acetate; and (v) the d-fenchone chemotype, with a content of 83.7% d-fenchone [48–50]. However, for the same plant material collected in the central regions of Benin, Alitonou et al. [27] identified only the thymol (63%) and carvacrol (51.1%) chemotypes. This variability in the chemical composition of essential oils of the same plant material could be due to many factors, including the bioclimate, soil composition, the harvesting period, the geographical location, the degree of maturity of the plant, the seasonal variation and even the plant genetic background [51].

The results from larvae bioassays using the laboratory colonies of *An. gambiae* s.s. showed that the essential oil of *A. pubescens* aerial parts was highly active (LC_50_ < 50 ppm) on the specimens of these strains according to the classification of Komalamisra et al. [52]. Larvae of both the Acerkis and Kiskdr strains were most susceptible to the essential oil (RR_50_ < 1). These results suggest that the essential oil had a promising larvicidal property with low LC_50_ values. The significant activity on the resistant strain over the reference susceptible Kisumu mosquitoes indicates that the essential oil does not affect one of the former target sites [*ace-1*^*R*^ and *kdr*^*R*^ (L1014F) alleles] represented in the corresponding strain. Therefore, it could be implied that its mode of action is different from that of pyrethroids, organophosphates and carbamates. However, in the context of the increasing insecticide resistance in natural mosquito populations, it will be interesting to investigate the bioactivity of this essential oil on field-collected larvae and field-caught adults.

Other biological activities of *A. pubescens* essential oil have been reported, including antioxidant [27, 49] and antibacterial [53] activities, but to date there has been no report on its mosquitocidal activity. In addition, there is still a lack of information on the bioinsecticidal property of the other plant species belonging to the genus *Aeollanthus*. However, previous studies have investigated the larvicidal activity of the essential oil of plants belonging to the same family (Lamiaceae). Tchoumbougnang et al. [54] showed that *Ocimun canumm*,* Ocimum gratissimu* and *Thymus vulgaris* had LC_50_ values of 201, 180 and 119 ppm, respectively, on field-collected *An. gambiae* larvae. These values are higher than those recorded in our study. This variation could be due to the difference in the chemical composition of the oils and the genetic background of the larvae strains used. Essential oils from other Lamiaceae species (*Plectranthus amboinicus* and *Plectranthus mollis*) have also been found to be active against *Anopheles stephensi* larvae, with a LC_50_ value of < 50 ppm [55, 56]. These findings suggest that essential oils from *Lamiaceae* plant species could be a potential source of environmentally eco-friendly mosquitocidal agents. In our study, the content of *A. pubescens* essential oil was dominated by monoterpenes (oxygenated and hydrocarbons), which accounted for 82.8% of the oil’s content. Other plants species with similar major constituents have been reported to be active against *An. gambiae* larvae*.* Ollengo et al. [57] reported that *Clausena anisata* essential oil, with 56.7% monoterpene content, possessed a potential larvicidal activity against *An. gambiae* (LC_50_ = 75.96 ppm) [57]. Also, Wangrawa et al. [58] demonstrated that *Lantana camara* essential oil, with 70.5% monoterpene content, resulted in differential larval mortalities on both the laboratory and the field strains of *An. gambiae*. The high proportion of monoterpenes in the essential oil could be correlated to the observed bioactivity. Indeed, many studies have reported the larvicidal effect of monoterpenes against mosquitoes strains [59–61]. Therefore, it would be interesting to evaluate further the toxicity of the monoterpenes isolated from the *A. pubescens* aerial parts on mosquito larvae in both laboratory and field trials.

Carvacrol is well known for its larvicidal property against *An. stephensi*, *Anopheles subpictus*, *Aedes aegypti*, *Culex quinquefasciatus* and *Culex tritaeniorhynchus* [62–65]. It will be interesting to evaluate further whether carvacrol, the main compound (51.1%) found within the monoterpenes in our *A. pubescens* oil extract, could be responsible for the observed activity against the *An. gambiae* larvae.

Essential oils are mixtures of volatile compounds and due to the antagonistic or synergistic phenomena, the bioactivity of the crude oil extract in some cases is lower or higher than those of the purified compounds. For example, Evergetis et al. [66] demonstrated that the larvicidal activity of the essential oil of *Origanum vulgare* against *Aedes albopictus* (LC_50_ = 30.1 ppm) is lower than that of its major component, the pure carvacrol (LC_50_ = 13.1 ppm), which accounts for 88.7% of the oil content. The same trend was noted with the leaf essential oil of *Coleus aromaticus*, which displayed lower toxicity than its major component carvacrol against *An. stephensi* larvae [67]. These findings open the perspectives for further investigations to evaluate the larvicidal efficacy of carvacrol in comparison to that of the crude oil extract. It is well known that monoterpenes from essential oils can act by absorption through the cuticle, via the respiratory tract or by ingestion via the gastrointestinal tract [68–71]. In addition, several monoterpenes have been reported to target primarily the cholinergic, octopamenergic and Gamma aminobutyric acid (GABA) neurosystems in insects [72]. One or a combination of these mechanisms might be the pathway of mortality induction by the *A. pubscens* oil. In this study, larvae of the reference resistant strain Acerkis larvae haboring the *ace-1*^*R*^ allele coding for the insensitive acetylcholinesterase enzyme was the most susceptible to our essential oil. This indicates that the essential oil overcomes the target site modification resistance mechanism and therefore appears to be a hopeful alternative tool for vector control programs.

Of the vector life-history traits, mosquito survival is strongly associated with malaria transmission intensity [73]. Thus, this study also investigated the effect of exposure to various doses of *A. pubescens* oil on the survival of *An. gambiae* adults*.* At doses 55 and 110 µg/cm^2^ of the essential oil, the lifespan of mosquitoes was 14 days maximum for the resistant Acerkis and Kiskdr strains. During this time period, mosquitoes might still be able to reproduce. Therefore, further studies are needed to assess the blood-feeding success, the fecundity and the fertility of mosquitoes following exposure. This could lead to the highlighting of putative detrimental effects of the essential oil exposure that could also hamper the vectorial competence of the mosquitoes. The lifespans of the three mosquitoes strains exposed to the pieces of net impregnated with 165 µg/cm^2^* A. pubescens* essential oil were significantly reduced. None of the three mosquito strains was able to survive after 72 h. This observation suggests that even resistant mosquitoes (Acerkis and Kiskdr strains) could not survive long enough to allow the extrinsic incubation period of the *Plasmodium* parasites if they ingested a gametocyte-infected blood meal.

Overall, the observed drastic reduction in daily survival of mosquitoes exposed to the oil treatment at 165 µg/cm^2^ might contribute to a reduction in vectorial capacity in a typical endemic setting and therefore to a reduction in parasite transmission according to the Ross–MacDonald model [74]. This is a promising finding for the management of the resistant malaria-transmitting vectors. Spray-type solution formulations could be manufactured for the development of botanical insecticides to be used in an integrated approach with the existing conventional vector control strategies. However, in our study, the results were obtained using mosquito strains in which only one resistance mechanism is present. It will be interesting to evaluate further the survivorship of the natural mosquito populations where several resistance mechanisms could coexist. The susceptibility of *Plasmodium* infection following exposure of the mosquito vector to essential oil is also another promising parameter to be evaluated.

During the experiment, we observed that the legs of mosquitoes detached from their bodies when exposed to the net coated with essential oil at 165 µg/cm^2^. To our knowledge, this phenomenon has not been observed with essential oils, but it is known to happen with insecticides of the pyrethroid group. However, such mechanism is so far unexplored. Possible neurotoxicity in insects could have been easily overlooked. Thus, investigations on the mechanisms by which the essential oil achieves its effect are urgently needed. The detachment of the mosquito legs suggests that the essential oil could interfere with the insect locomotor system and even the nervous system, leading to death in the following days.

## Conclusion

The findings of the present study pave the way to the development of a new and safer natural insecticide against malaria mosquito vectors. The *A. pubescens* essential oil was shown to be an efficient larvicide and adulticide against the malaria vector *An. gambiae.* This widens the perspectives for implementing sustainable control of mosquito populations that are resistant to current existing synthetic insecticides. Larval and adult vector control with the essential oil could be considered in an integrated fashion to the existing malaria control strategies. Further studies are needed to help in designing an *A. pubescens* essential oil formulation that would potentially increase its efficacy on *An. gambiae* and its cost-effectiveness.

## Supplementary Information

**Additional file 1: Table S1.** Larval bioassay data. **Table S2.** Adult mosquito knockdown time following exposure to the net pieces impregnated with the essential oil. **Table S3.** Adult mosquito daily mortality data following exposure to the net pieces impregnated with the essential oil.

## Data Availability

All data generated or analysed during this study are included in this published article and Additional file 1.

## Abbreviations

ACTs: Artemisinin-based combination therapies
DDT: Dichlorodiphenyltrichloroethane
GC–FID: Gas chromatography coupled with flame ionisation detection
GC–MS: Gas chromatography coupled with mass spectrometry
i.d.: Internal diameter
IRS: Indoor residual spraying
ITNs: Insecticides treated nets
*kdr*^*R*^: Knockdown resistance allele
KDT_50_: Knockdown times for 50% of adult mosquitoes
KDT_95_: Knockdown times for 95% of adult mosquitoes
L1014F: Leucine substitution by phenylalanine at position 1014
LC_50/95_: Lethal concentrations (50 and 95% mortality, respectively)
LRT: Likelihood ratio test
RI: Relative retention indices
RR_50_: Resistance ratio
WHOPES: World Health Organisation Pesticide Evaluation Scheme
